# No need to discontinue hepatitis C virus therapy at the time of liver transplantation

**DOI:** 10.1371/journal.pone.0211437

**Published:** 2019-02-22

**Authors:** Catarina Skoglund, Martin Lagging, Maria Castedal

**Affiliations:** 1 The Transplant Institute, Department of Surgery, Sahlgrenska University Hospital, Gothenburg, Sweden; 2 Institute of Clinical Sciences, Sahlgrenska Academy, University of Gothenburg, Gothenburg, Sweden; 3 Department of Infectious Diseases/Virology, Sahlgrenska University Hospital, Gothenburg, Sweden; 4 Institute of Biomedicine, Sahlgrenska Academy, University of Gothenburg, Gothenburg, Sweden; Nihon University School of Medicine, JAPAN

## Abstract

**Objectives:**

Direct antiviral agents (DAA) has dramatically improved the therapy outcome of hepatitis C-virus (HCV) infection, both on the waiting-list and post liver transplantation (LT). DAA are generally well-tolerated in patients with mild to moderate liver and kidney failure, but some DAAs are contraindicated in patients with severe dysfunction of these organs. Today there are few studies of peri-LT DAA use and treatment is commonly discontinued at the time of LT. We report here our experience of DAA therapy given continuously in the perioperative LT period in a real-life setting in Sweden.

**Material:**

In total 10 patients with HCV-cirrhosis, with or without hepatocellular carcinoma, and a median age of 60.5 years (range, 52–65) were treated with DAAs on the waiting list for LT, and continued in the early postoperative period without any interruption, on the basis of not having reached a full treatment course at the time of LT. Sofosbuvir and a NS5A inhibitor with or without ribavirin, or sofosbuvir and ribavirin only, were given. The distribution of genotypes was genotype 1 and 3, in 4 and 6 patients, respectively. Six of the 10 patients had previously been treated with IFN-based therapy.

**Results:**

There were no adverse events leading to premature DAA discontinuation. All recipients achieved a sustained viral response 12 weeks after end-of-treatment (SVR12). At the time of LT the median MELD-score was 16.5 (range 7–21), CTP-score 9.0 (range 5–10), creatinine 82.5 μmol/L (range 56–135, reference 60–105), bilirubin 33 μmol/L (range 16–79, reference 5–25) and PK-INR 1.5 (range 1.1–1.8). The median duration of DAA therapy was 60 days (range 18–132) pre-LT, 54 days post-LT (range 8–111 days) and in total 15.5 weeks (range 12–30 weeks).

**Conclusion:**

Interferon-free DAA therapy of HCV-infection given in the immediate pre- and post-operative LT period is safe, well-tolerated and yields high SVR rates.

## Introduction

Chronic hepatitis C virus (HCV) infection affects an estimated 71 million people in the world with fatal consequences in approximately 400,000 persons per year [1]. About 20% of the chronically infected patients develop cirrhosis or liver cancer, necessitating liver transplantation (LT) within 20 years postinfection. After LT recurrence is universal in patients who are viremic at the time of the operation. HCV-infection in LT recipients has an accelerated progress and 20–30% will develop cirrhosis within 5-years post-LT if not treated [2, 3, 4]. The graft survival in HCV-recipients has shown to be significantly lower than in recipients transplanted for other liver diseases [3, 5, 6].

Tolerance of the previous standard-of-care (SOC) treatment, pegylated-interferon (peg-IFN) and ribavirin (RBV), in cirrhotic patients as well as LT recipients was suboptimal due to severe side-effects such as infections and increased risk of rejection, leading to frequent cessation of therapy [7]. Furthermore, peg-IFN and RBV combination therapy in LT patients yielded SVR rates as low as 20–45%, with only 15–30% of genotype 1 infected recipients achieving SVR [3, 8]. In the new era of IFN-free therapy with DAA, outcome has improved dramatically both on the waiting list for LT and post-LT due to fewer side-effects [9–12].

However, some patients on the waiting list will undergo LT before receiving a full DAA treatment course, and therapy is commonly stopped at the time of LT because of fear of unrecognized DAA-associated complications in the immediate postoperative days, as this time period rarely has been included in larger therapeutical trials.

Thus, the aim of the current study was to evaluate the safety and efficacy of DAA therapy given without interruption in the peri-LT period in a real-world setting. Our findings confirm a high tolerability and efficacy of DAA treatment in this vulnerable situation with fragile patients undergoing LT.

## Patients and methods

### Patients

All liver transplanted patients >18 years of age with chronic hepatitis C infection (HCV) who received DAA therapy continuously in the pre- and post-LT period, between April 2013 to December 2016, at the Transplant Institute, Sahlgrenska University Hospital, Gothenburg, Sweden, were included in this retrospective study. All patients were transplanted due to end-stage liver disease and/or hepatocellular carcinoma (HCC). The retrospectively collected data were not considered by the ethical committee (diary number 340–16), the Swedish Medical Products Agency, or any other institutions to violate personal integrity, and accordingly the ethical committee did not require any written or oral consent. No special recording concerning patient consent was performed for this retrospective study as it was not required by the Gothenburg Ethical Committee (Regionala etikprövningsnämnden i Göteborg). This study is conformed according to the ethical guidelines of the 1975 Declaration of Helsinki.

### Treatment

Patients received IFN-free sofosbuvir-based therapy, in combination with daclatasvir, daclatasvir and RBV, ledipasvir and RBV or RBV only, respectively. The DAA-regimens were chosen based on two different situations. In the very early study period, around 2013, the only DAA available was sofosbuvir and this was the only drug of choice. Sofosbuvir was combined with ribavirin in these cases. In later time periods when NS5A-inhibitors had become available, we chose to use NS5A-inhibitors in combination with sofosbuvir ± ribavirin. This choice was based on the potential interactions between HCV protease inhibitors and calcineurin inhibitors, especially ciclosporin, making this alternative less attractive. Thus, HCV protease inhibitors were avoided if possible.

The DAA treatment was initiated when patients were on the waiting list for LT and continued at the day of surgery as well as directly post-LT. The baseline immunosuppression given post-LT was a combination of tacrolimus (TAC) and mycophenolate mofetil (MMF).

### Methods

All liver transplant candidates and all liver transplant patients are very rigorously followed according to our liver transplantation protocol, which includes daily monitoring of various blood parameters, control of immunosuppressive levels, in addition to thorough clinical assessments.

Genotype analysis was performed prior to DAA-therapy (Table 1). HCV RNA quantification was performed before initiation of DAA therapy while on the waiting list, and thereafter intermittently until LT. Posttransplant HCV RNA levels were measured at end of DAA treatment, and during follow-up 4, 12 and 24 weeks after DAA cessation. End of treatment viral response (ETR) was defined as undetectable HCV RNA at the time of treatment discontinuation, and sustained viral response (SVR) as undetectable HCV RNA 12 weeks after end-of-treatment. HCV RNA analyses were performed using Roche COBAS TaqMan, with a limit of detection of <15 IU/mL.

**Table 1:** Patient demographics preoperatively at the day of liver transplantation. Figures are presented as numbers and medians (range), respectively.

	Baseline DAA	Baseline LT
**Numbers (n)**	10	10
**Age (yrs)**	60.5 (52–65)	60.5 (52–65)
**Sex (M/F)**	8/2	8/2
**BMI**		26.4 (21.4–29)
**HCV RNA negativity**	0	10
**Genotype (n)**		
1a	3	3
1 (subtyping missing)	1	1
3a	6	6
**Hemoglobine g/dL**	120.5 (88–151)	122 (86–139)
**Platelets x 10**^**9**^**/L**	88.5 (40–129)	100 (41–225)
**Creatinine μmol/L**	72.5 (49–122)	82.5 (56–135)
**ALT μkat/L**	1.26 (0.58–2.56)	0.475 (0.17–0.97)
**AST μkat/L**	1.32 (0.84–5.6)	0.635 (0.35–1.3)
**Bilirubin μmol/L**	36.5 (11–120)	33 (16–79)
**PK-INR**	1.35 (1.1–1.9)	1.5 (1.1–1.8)
**Albumine G/L**	32 (28–42)	33 (24–41)
**Ascites**	7 (3 grade 1, 4 grade 2 or 3)	4 (grade 2 or 3)
**HE at LT**	2 (grade 1 and 4)	1 (grade 1)
**MELD-score**	12 (7–24)	16.5 (7–21)
**CTP-score**	9 (5–12)	9 (5–10)
**DAA-therapy**		
Sofosbuvir + Daclatasvir	0	4
Sofosbuvir + RBV	0	3
Sofosbuvir + Daclatasvir + RBV	0	2
Sofosbuvir + Ledipasvir + RBV	0	1
**Cause of Liver transplantation**		
HCC	4/10	
HCC + decompensated cirrhosis	3/10	
Decompensated cirrhosis	3/10	

Abbreviations: BMI, Body Mass Index; ALT, Alanine aminotransferase, AST, Aspartate aminotransferase; INR, International Normalized Ratio; HE, Hepatic encephalopathy; LT, Liver transplantation; MELD, Model for End-stage Liver Disease; CTP, Child-Turcotte-Pugh score; DAA, Direct Antiviral Agents; RBV, Ribavirin; HCC, Hepatocellular carcinoma.

Blood parameters including full blood cell count, electrolytes, creatinine and biochemical response to DAA therapy were analyzed at baseline, week 1, 2, and 4 on DAA treatment and hereafter every 4^th^ week until LT. At baseline, every 4^th^ week until LT as well as preoperatively at the day of LT, MELD- (Model for End-stage Liver Disease) and CTP (Child-Turcotte-Pugh)-scores were calculated (Table 1). Postoperatively, full blood cell count, electrolytes including creatinine, liver function tests and C-reactive protein (CRP) were taken daily during the first week and thereafter as clinically indicated. There was a high alertness for any medical side-effects or drug interactions.

The criteria used to interrupt or discontinue DAA-therapy was based on a 2-step decision tree. First, every complication pre-, peri- and post-liver transplantation was thoroughly assessed and, when needed, adjusted/treated according to our standard of care procedures. Secondly, if the patient deteriorated despite necessary interventions/medical changes DAA-cessation was seriously considered.

## Statistical analysis

This study is a retrospective, descriptive cohort study. Categorical data are presented as numbers and percentages and numerical data as medians and range.

## Results

### DAA therapy

**Fig 1:**
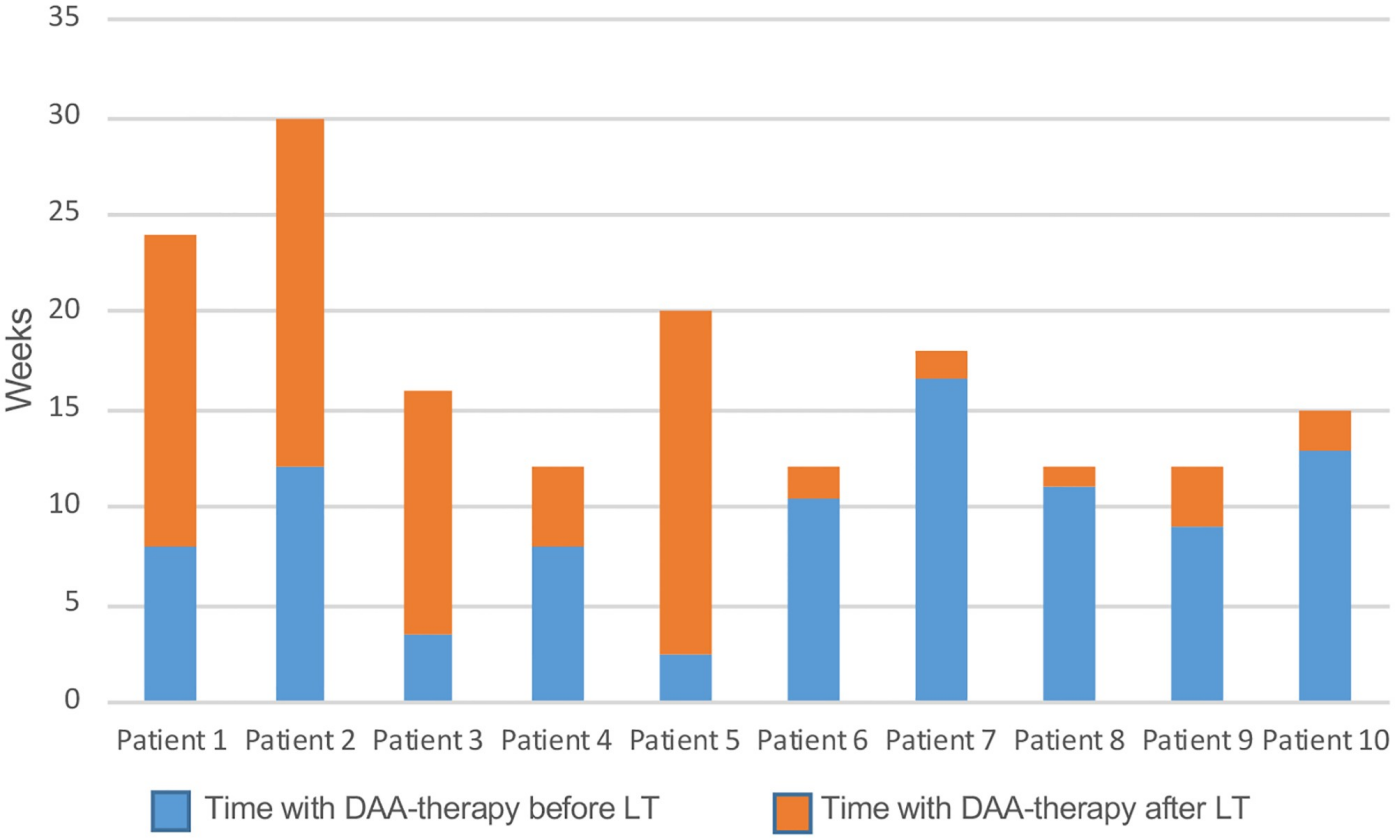
DAA treatment pre- and post-LT. DAA-treatment before and after liver transplantation. Blue and orange bars represent pre- and post-transplant therapy length, respectively.

Sixty-eight adult HCV-patients were liver transplanted between May 2013 and December 2016. Of these patients 31 were cured by DAA treatment while on the waiting list for LT, 17 initiated DAA therapy post-LT, and 10 recipients were treated before wait-listing or were planned for HCV-therapy post-transplant at their local hospitals. The remaining 10 (8.6%) patients composing our study cohort, initiated DAA when on the waiting list and continued therapy, without interruption, on the day of LT and early post-operative. The 10 study patients received sofosbuvir-based treatment, 4 in combination with daclatasvir, 3 together with RBV only, 2 with daclatasvir and RBV and 1 patient with ledipasvir and RBV. In all, 6 of 10 patients received RBV. The DAA therapy duration on the waiting list was in median 60 days (range 18–132) and continued in median for 54 days post-LT (range 8–111) (Fig 1). The total treatment duration was 15.5 weeks (range 12–30).

### Recipient demographics

Patient demographics preoperatively at the LT-day are reported in Table 1. Eight patients were men and 2 were women, with a median age of 60.5 (range 52–65) years. All patients were Caucasians and had HCV-induced liver cirrhosis. Before start of DAA-therapy HCV-genotype was analyzed and genotype 3a infection was noted in 6 (60%) patients and genotype 1 in 4 (40%) patients, where 3 had subtype 1a and in one patient subtyping was not available. Seven of the 10 patients had coexisting hepatocellular carcinoma (HCC) confirmed in the explant. Four of these patients were anti-HBc positive, but HBsAg negative, consistent with an inactive hepatitis B virus-infection. Three patients had previous alcohol dependence and 2 were insulin dependent diabetics.

### Virological response

**Fig 2:**
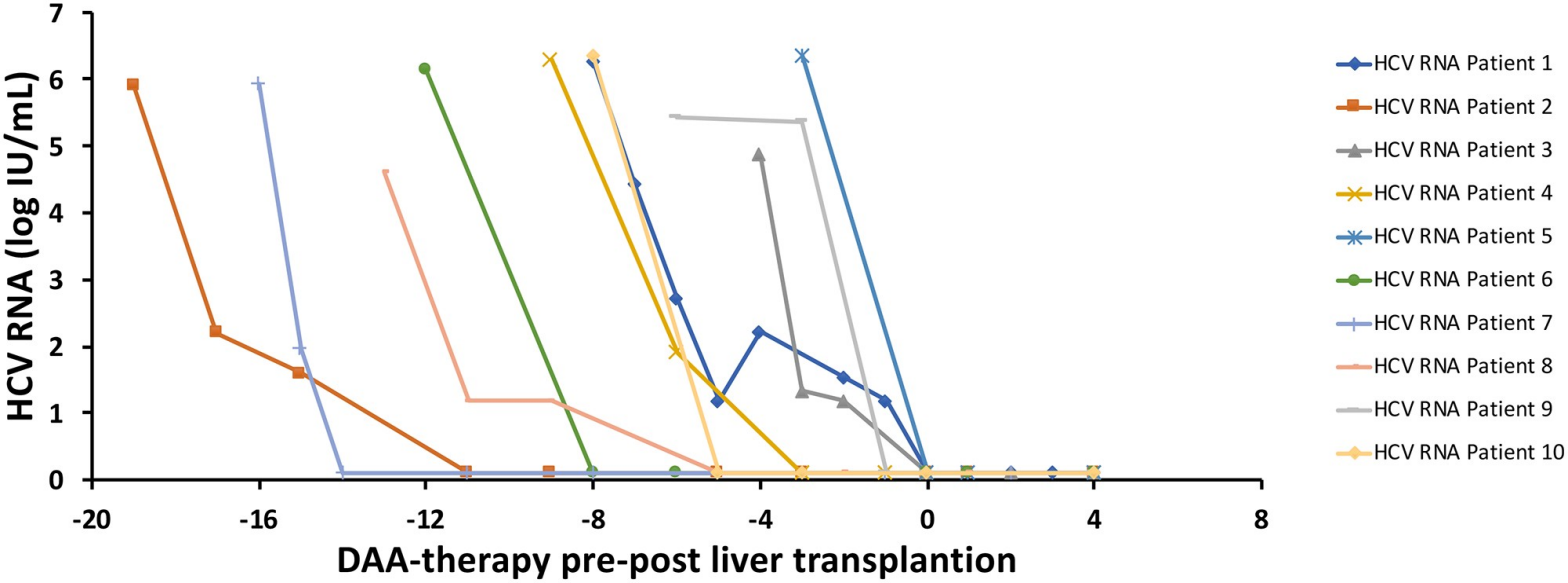
HCV-RNA levels pre- and post-LT. The individual HCV RNA levels at start of DAA-therapy, at the day of liver transplantation as well as up to 4 weeks post-transplant, i.e., the study period.

All patients had an on treatment virological response (OTVR) and undetectable HCV RNA in serum at the time of LT. HCV-RNA levels for each patient both before and after liver transplantation are illustrated in Fig 2. Serum HCV RNA had been undetectable for ≥4 weeks in 5 patients and <4 weeks in 5 patients before the LT. All 10 patients (100%), achieved an end-of-treatment response (ETR), as well as a sustained viral response week 12 (SVR12).

### Safety and tolerability

The adverse events reported during the day of LT as well as during the first postoperative month, divided into two time periods, are summarized in Table 2. There were no deaths in this study. No patient had to discontinue DAA treatment due to side-effects, and none of the patients experienced any rejection episodes.

**Table 2:** Safety and tolerability of DAA in the immediate perioperative period. Adverse events (AE) are shown for the day of liver transplantation and for two different time periods during the first postoperative month. The number of patients presenting each AE are presented.

AE	LT-day	Post-LT day 1–7	Post-LT day 8–30
**Death**	0	0	0
**DAA cessation due to AE**	0	0	0
**Rejection episodes**	0	0	0
**Laboratory abnormalities:**			
Hgb <90g/dL	1	3	5
WBC <3.5x10^9^ cells/L	0	1	1
Platelets <50x10^9^ cells/L	2	2	1
Total bilirubin>2.5xULN	2	4	0
INR>2x ULN	0	0	0
**Renal dysfunction**	3	2	1
**Haemodialysis/CRRT**	1	1	0
**Ascites**	4	0	0
**Pleural effusion/** **Pneumothorax**	1	1	0
**HE/confusion**	1	1	1
**Abdominal bleeding:**	2	0	0
from VCI anastomosis	1		
from lumbal vein	1		
**Peroneal nerve palsy**	1	1	0
**Epileptic seizure/PRES**	0	0	2
**Atrial fibrillation**	0	1	0
**Pruritus**	0	0	1
**Alopecia**	0	0	1
**Nausea**	0	0	1

Abbreviations: DAA, Direct acting antivirals; Hgb, Hemoglobine; WBC, White blood cell count; INR, International Normalized Ratio; ULN, Upper limit of normal; CRRT, Cintinous renal replacement therapy, HE, Hepatic encephalopathy; VCI, Vena cava inferior; PRES, posterior reversible encephalopathy syndrome.

Preoperatively at the LT-day one patient had hemoglobine <90g/dL deemed related to severe liver disease, neoadjuvant therapy with sorafenib for HCC and RBV therapy. During the first and second postoperative period, 3 and 5 patients, respectively, were anemic (<90g/dL) related to surgical bleeding (2 pts), RBV and immunosuppressive therapy. One patient developed neutropenia (<3.5x10^9^) post-LT related to hypersplenism and MMF therapy. Two patients had severe thrombocytopenia (<50x10^9^ cells/L) related to hypersplenism while on the waiting list, before DAA initiation, with low levels also at the LT-day aggravated further during the first postoperative week. Two patients had bilirubin >2.5xULN at the LT-day judged to be caused by chronic liver disease and RBV therapy. These two patients plus another two had hyperbilirubinemia during the first post-LT week assessed related to abdominal bleeding (2pts), RBV treatment and delayed graft function (1pts), respectively. The two patients with abdominal bleeding had to be re-operated within 2 days post-LT.

Renal dysfunction was seen in 3 patients preoperatively (Table 2), one having severe hepatorenal syndrome (HRS) needing a few hours of CRRT directly postoperative. Another patient with pre-LT renal impairment required CRRT/hemodialysis during the first post-LT week judged related to the additive negative impact of the LT trauma, the abdominal bleeding and reoperation as well as delayed liver graft function, on renal function. Ribavirin had to be stopped, but the DAAs (SOF, DCV) were continuously given, initially via a nasogastric tube. The renal function resolved during the first post-LT month. This female patient, requiring CRRT and reoperation shortly after LT, was intermittently confused during the first 3 post-LT weeks, but could be discharged to the referral hospital 25 days post-LT. However, one week later, corresponding to one month post-LT and to the time of scheduled DAA cessation, she developed an epileptic seizure. This seizure and the previous post-LT episodes with confusion was assessed as possibly related to tacrolimus induced posterior reversible encephalopathy syndrome (PRES) [13], and she was therefore switched to cyclosporine therapy. After this change in immunosuppression no further seizures were reported. Performed MRI scan of the brain as well as an EEG showed normal findings.

The other female patient in this small study developed severe CNS symptoms starting one month post-LT corresponding to 3 weeks post DAA stop (Table 2). She suddenly experienced an epileptic seizure followed by left hemiparesis and 3 weeks later yet another seizure developed. Repeated CT and MRI scans of the brain showed signs of cerebral vasculitis and bleeding both frontally and parietally and there was also a typical edema in the occipital lobe compatible with PRES [13], partially suspected to be related to tacrolimus treatment and she was therefore switched to everolimus-based immunosuppression. Hereafter no further seizures was noted. Her left hemiparesis slowly improved.

## Discussion

To our knowledge, the present study is the first to confirm that DAA therapy is safe and well-tolerated also in severely ill LT-candidates in the immediate peritransplant period and yields high SVR12 rates. Direct acting antivirals are generally well tolerated in patients treated on the waiting list for LT as well as a few months after LT when the clinical situation often is relatively stable. However, impaired liver and renal function as well as potential drug interactions, in particular with the immunosuppressive medications, may limit the DAA treatment options early after LT [14]. In the perioperative setting LT-recipients are especially vulnerable to medical side-effects due to the operative trauma. Despite this none of the patients enrolled in the present study had to discontinue their DAA-medications. Although not an officially approved indication, two patients still requiring mechanical ventilation during the first postoperative days were given their DAAs via a nasogastric tube after being crushed, which was feasible as the tubes were thoroughly flushed with water afterwards.

Interactions between the immunosuppressive drugs and DAAs are well documented, e.g. the interactions between calcineurin inhibitors (CNI) and protease inhibitors [14]. In this study, only the nucleotide analogue sofosbuvir in combination with a NS5A inhibitor with or without ribavirin was used, decreasing the risk of interactions with CNIs as well as with the mTOR-inhibitor, everolimus. However, the immunosuppressive trough levels were, as always, closely monitored in the early postoperative period, and doses adjusted as needed. The immediate post-LT period is often an instable period with changes in both liver and renal function and it is therefore less feasible to study the effect of DAA co-administration in this period. An improved or temporarily worsened liver function directly post-LT, e.g. in the case of delayed graft function, can in combination with the inherited capacity of the CYP-450 system in the new liver graft and any potential renal dysfunction, impact on both DAA and CNI pharmacokinetics. Clinicians should therefore carefully choose DAA treatment options for patients on the waiting list for LT. The present study with sofosbuvir- and NS5A-based antiviral therapy showed no obvious DAA related complications in the early post-LT period in this fragile patient cohort with mostly Childs B and C cirrhosis pre-LT and mild to severe renal impairment, including two patients on CRRT the first postoperative week.

In the current study none of the patients had a rejection during the post-LT study period (Table 2). This low rejection incidence [15] may be secondary to the close monitoring of the CNI target trough levels adopted during the first postoperative month or simply a result of the small sample size.

Both our female study patients developed seizures approximately one month post-LT. One of them was intermittently confused during the first 3 postoperative weeks. She had delayed liver graft function, mild hypertension, renal failure and 4 weeks post-LT at the day of scheduled DAA stop, an isolated seizure. The cerebral symptoms may have been caused by a posterior reversible encephalopathy syndrome (PRES) [13], which is known to be triggered by hypertension, renal impairment, infections, and immunomodulating drugs. She was successfully switched to cyclosporine-based immunosuppression with clinical improvement within a week.

The other female patient also had a seizure at 1 month post-LT judged by neurologists and the transplant team to be caused by cerebral bleeding and vasculitis, complicated a few weeks later by PRES. We therefore chose to change the CNI immunosuppression also in this patient with subsequent favorable results regarding the PRES symptoms. Any relation between PRES and DAA therapy seems unlikely since the DAAs were stopped 3 weeks before the first cerebral symptoms.

In our study 7 of 10 patients had hepatocellular carcinoma (HCC) as one of the indications for LT. None of them had recurrence of their HCC during DAA treatment or during the study follow-up period. There have been many contradictive reports on whether DAA-therapy increases the incidence of HCC-recurrence [16] as well as de novo HCC [17]. We found no negative impact on HCC recurrence by DAA therapy since all 7 patients with pre-LT HCC had no radiological signs of HCC recurrence after a median follow-up of 31.5 months (range 3–52 months) post-DAA-stop and 32 months (range 0–52 months) post-LT. Furthermore, the 3 non-HCC patients had no clinical signs of HCC occurrence at the 1-year post-LT follow-up.

In one of our earlier studies of DAA therapy in a post-LT cohort, the SVR12 rate was 97.8% [18]. This good result in post-LT recipients is in agreement with earlier reports and with the results of our current small cohort having 100% SVR12 and SVR24, maybe as a result of no need for premature DAA discontinuation.

Some reports suggest that all patients, irrespective of their HCV genotype, should be treated immediately after LT to prevent recurrent hepatitis C and fibrosis progression in the graft [19, 20]. Our results of high tolerability of DAAs also in the peri-LT period supports continuing the DAA therapy, started while patients are on the waiting list, also in the immediate post-LT period. According to the study by Curry et al [21] the best predictor of post-LT SVR is undetectable serum HCV RNA for ≥4 weeks pre-LT, supporting discontinuation of DAA at the time of LT in these patients, while continuing DAAs without any interruption postoperatively, in those with <4 weeks of HCV RNA negativity. This "4-week-rule" has since early 2016 been incorporated into the Swedish HCV guidelines and implemented with good results [22]. Due to this late implementation of the "4-week rule" during the study period, 5 of 10 patients received continued DAA therapy for a median of 12 days post-LT (range 8–56) despite being HCV RNA negative for ≥4 weeks before LT.

A future shorter treatment alternative, especially in those with burnt-out cirrhosis or cirrhosis in combination with HCC and no chance for curative pre-LT DAA therapy, may be to initiate DAA therapy when the patient is transferred to the transplant center for the LT-operation, and to continue DAA therapy directly post-LT, without interruption, for a short period of time, e.g. 4–8 weeks. This strategy may be sufficient to eradicate the HCV-infection when combined with explanting the cirrhotic HCV-infected liver, and is strenghtened by our study, but warrants further investigation.

Although our data points towards excellent outcome following DAA therapy in the immediate peri-LT setting, it is important to highlight the limitations of the study, e.g., the small sample-size, few HCV genotypes represented (although 6 of our patients had genotype 3 which is the hardest to cure), and no use of protease inhibitors. Further studies are necessary to confirm that DAAs given in the immediate peri-LT period are safe, well-tolerated and effective in this vulnerable patient category. If our results are confirmed expensive re-treatments after LT, due to premature discontinuation of DAA therapy at time of operation, may be avoided. Furthermore, the challenging task to differentiate between rejection and active HCV-infection in posttransplant liver biopsies will be an eliminated problem.

In conclusion, there is no need to discontinue DAA therapy at the time of LT in patients given DAA while on the waiting list. Antiviral therapy can, if carefully selected, safely be continued without interruption in the immediate perioperative period. This strategy may be a well-tolerated, shorter and less expensive HCV treatment option yet leading to high SVR-rates.

## Supporting information

S1 Dataset (XLSX)
